# Assessment of the Clinical Condition and Way of Patients’ Nutrition before and after Laparoscopic Sleeve Gastrectomy

**DOI:** 10.3390/nu15030514

**Published:** 2023-01-19

**Authors:** Marta Jastrzębska-Mierzyńska, Lucyna Ostrowska, Katarzyna Witczak-Sawczuk, Hady Razak Hady

**Affiliations:** 1 Department of Dietetics and Clinical Nutrition, Medical University of Bialystok, 15-054 Bialystok, Poland; 2 1st Clinical Department of General and Endocrine Surgery, Medical University of Bialystok, 15-276 Bialystok, Poland

**Keywords:** obesity, sleeve gastrectomy, body composition, nutrition, blood biochemical parameters

## Abstract

One of the most commonly performed bariatric procedures is the laparoscopic sleeve gastrectomy (LSG). It is highly effective in reducing body weight, but it carries the risk of developing nutritional deficiencies and their consequences. The aim of the study was to determine the clinical condition of obese patients after LSG in terms of nutritional status, metabolic disorders, and way of nutrition. Thirty participants (15 women and 15 men) took part in the study. A statistically significant reduction in the total body fat mass (women by 37.5% *p* < 0.05, men by 37.06% *p* < 0.05) and total fat free mass (women by 10% *p* < 0,05, men by 12.5% *p* < 0.05) was demonstrated 6 months after LSG. Moreover, insufficient protein intake has been shown in over 73% of women and 40% of men. Before and 6 months after LSG, insufficient intake of calcium, magnesium, potassium, folate, vitamin D, and iron was observed. Six months after the LSG, significant decreases of fasting glucose (*p* < 0.05), insulin (*p* < 0.05), TG (*p* < 0.05), and AST (*p* < 0.05) concentrations, were observed in both groups. Optimization of nutrition in order to prevent nutritional deficiencies and their complications is a key element of the therapy of obese patients treated surgically.

## 1. Introduction

Obesity is a chronic disease characterized by abnormal or excessive accumulation of fat tissue [1]. The frequency of occurrence of obesity, both in Poland and worldwide is steadily increasing. According to the 2019 data from Center of Public Opinion Research in Poland, 21% of adults suffer from obesity [2]. According to Eurostat, this index in 2019 was 19% [3].

Preservative treatment of obesity, especially morbid obesity, is often inefficient and the only alternative method is surgical treatment. In addition to significant and permanent reduction of excessive body weight, it also leads to remission of co-morbidities, such as type 2 diabetes, hypertension, lipid disorders, and non-alcoholic fatty liver disease [4].

Sleeve gastrectomy is one of the most frequently performed bariatric procedures in Poland and worldwide. It includes resection of a large part of the stomach, reducing its volume to the point when only small portions of food may be consumed by the patient. Significant reduction of energy and nutrient intake as a result of stomach volume reduction may cause nutritional insufficiencies and malnutrition.

It is indisputable that sleeve gastrectomy adversely affects the status of nutrition of patients, as well as the composition of their bodies and durability of body weight reduction. It should be highlighted that nutritional deficiencies also occur in obese patients before the surgery. According to available data, those most commonly diagnosed are iron, vitamins (B_12_, D, B_1_), and folic acid deficiencies [5,6,7,8,9,10]. Therefore, it seems necessary to provide dietitian care to patients, both before and after bariatric surgery. Periodic dietary visits enable regular nutritional education of patients, assessment of their diet and nutritional status, and implementation and modification of nutritional therapy. Optimization of patient nutrition seems to be one of the key elements influencing the effectiveness of bariatric treatment.

The aim of this study was a 6-month retrospective assessment of the clinical status of obese patients after laparoscopic sleeve gastrectomy in terms of nutrition, nutritional status, and metabolic disorders. The results of the analysis will enable balancing of the diet of morbidly obese patients qualified for surgical treatment in order to optimally prepare them and compensate for nutritional deficiencies which may influence the effectiveness of treatment and the health of patients after the surgery.

What is more, the study will reveal which nutrients patients lack in particular periods after LSG, which is necessary in planning dietary treatment and including appropriate supplementation.

## 2. Materials and Methods

The study included 30 obese patients (15 women and 15 men) qualified for laparoscopic sleeve gastrectomy. The procedure was performed in the 1st Clinical Department of General and Endocrine Surgery, Medical University of Bialystok. The inclusion criteria were age (18–64 years), BMI ≥ 40 kg/m^2^ or BMI ≥ 35 kg/m^2^ with two co-morbidities of obesity. The exclusion criteria were gastrointestinal cancers, severe cardiorespiratory failure, and pregnancy.

The study conducted four visits: a preliminary visit (1 day before the surgery) and control visits (1, 3, and 6 months after the surgery). On each visit, anthropometric measurements, body composition, and intake of energy and selected nutrients were assessed in the Department of Dietetics and Clinical Nutrition, Medical University of Bialystok. In addition, blood was collected for laboratory tests in the medical laboratory of the Medical University of Bialystok Clinical Hospital.

This study obtained an approval of the Bioethics Committee of the Medical University of Bialystok, Poland (No. R-I-002/481/2017). Each patient gave informed consent to participate in the study.

### 2.1. Operative Procedure and Postoperative Management

Sleeve gastrectomies were performed laparoscopically by a panel of surgeons including one operator and two assistants. The operations were performed with the patient in Fowler’s position at approximately 30° by using 5 trocars: 1 × 13 mm, 1 × 10 mm, 3 × 5 mm. The surgery started with creation of pneumoperitoneum. Once the intraperitoneal pressure reached 12 mmHg CO_2_, the trocars were introduced to insert laparoscope and surgery tools. The stomach was dissected along the greater curvature. Then, a 36-French Bougie was inserted into the stomach and served as a measure to create the new stomach volume. The stomach was cut off with the use of staplers. The staple line started approximately 4 to 6 cm from the pylorus and continued up to the angle of His. At the end of the surgery, the leak test was performed with the use of methylene blue.

Fasting was necessary for the first postoperative day. On the second postoperative day, drinking water was allowed. When biochemical tests such as complete blood count and *C*-reactive protein (CRP) were negative, patient started clear liquid diet which usually takes place on the third postoperative day. A liquid diet was usually continued for 2 weeks. If no complications occurred, a regular diet was implemented 2 weeks postoperatively.

When a patient is diagnosed with gastric leak, a self-expanding esophageal-gastric prosthesis is implemented by the endoscopic team.

### 2.2. Anthropometric Parameters and Body Composition Analysis

Anthropometric measurements were performed in patients wearing only light clothes and without shoes. Body weight (with an accuracy of 0.1 kg) and body height (with an accuracy of 0.5 cm) were determined using RADWAG WPT 100/200 OW scale with stadiometer. Body mass index (BMI) was calculated based on weight and height.

Percentage of excess weight loss (%EWL) was calculated for each patient 1, 3, and 6 months after LSG. %EWL was calculated using the formula: %EWL = (preoperative weight − current weight)/(preoperative weight − ideal weight) × 100 [11]. The ideal body weight was calculated from the Lorentz formula, where: ideal body weight (women) = height (cm) − 100 − ((height (cm) − 150)/2); Ideal body weight (men) = height (cm) − 100 − ((height (cm) − 150)/4) [12].

Body composition and resting metabolic rate (RMR) were measured using bioelectrical impedance by MALTRON BioScan 920-2 analyzer (Maltron International Ltd., Rayleigh, UK).

Changes in anthropometric measurements and body composition 1, 3, and 6 months after LSG in the group of women and men are presented in Table 1.

### 2.3. Blood Biochemical Parameters Analysis

Laboratory tests were performed to determine the following serum levels: fasting glucose, fasting insulin, total cholesterol, LDL cholesterol fraction, HDL cholesterol fraction, triglycerides and aminotransferases: alanine (ALT) and aspartate (AST).

Blood samples were collected and tested according to standard laboratory protocols applied by the hospital for routine analysis. Homeostasis model assessment of insulin resistance (HOMA-IR) was also calculated for each patient using the formula: HOMA-IR = (fasting insulin (mIU/L) × fasting glucose (mg/dL))/405. The percentage of patients with the results of individual biochemical tests above the reference values (and in the case of HDL-cholesterol below the reference values) in each of the periods was assessed.

### 2.4. Dietary Assessment

Dietary assessment was conducted applying 3-day 24-hour diet recall before the procedure as well as 1, 3, and 6 months after the procedure. The energy and nutritional value of the patient’s diet was determined using the DIETA 5.0 software. The assessment of the supply of nutrients did not include protein, vitamin, and mineral supplementation.

Furthermore, the percentage of patients in which the supply of particular nutrients was below the norm for the Polish population established by the National Institute of Public Health [13] was assessed. The RDA (Recommended Dietary Allowance) standard was adopted for protein, vitamins B_1_, B_6_, B_12_, folates, calcium, iron, magnesium, and zinc, and the AI (Adequate Intake) standard for fiber, vitamin D, and potassium.

In accordance with the Enhanced Recovery After Surgery (ERAS) Society recommendations regarding optimal protein intake after LSG, we administered 60 g per day [14]. We considered the normal intake of carbohydrates before surgery and 6 months after LSG to be 130 g/day. In the 1st and 3rd month after surgery, patients should intake at least 50 g of carbohydrates per day [13,15].

### 2.5. Statistical Analysis

Statistical analysis of the data was performed using Statistica version 13.3 (StatSoft Polska). The Shapiro–Wilk test was used to check the normality of the variable distribution. The data obtained 1, 3, and 6 months after the surgery were compared with the data obtained before the surgery. The non-parametric Mann–Whitney U test for two independent samples was used to compare the results in particular periods. Results with *p* < 0.05 were considered statistically significant.

## 3. Results

### 3.1. Anthropometric Parameters and Body Composition Analysis

Table 1 presents anthropometric measurements and body composition of women and men before the surgery as well as 1, 3, and 6 months after the surgery. The median age of women was 36 years and of men—42 years. In the 6-month follow-up, body mass, BMI, total body fat mass (FM), and fat free mass (FFM) were significantly reduced in both study groups.

The median EWL, 6 months after the procedure in the group of women was 49.5% and in the group of men—47.6% and it was significantly higher in comparison to the values registered 1 month after LSG. Six months after the procedure, EWL ≥ 50% was achieved by 40% of the examined women and 53.3% of examined men. In the 6-month observation, we showed a significant reduction in total body fat mass by 37.5% in the group of women and 37% in the group of men, as well as reduction in fat free mass by 10% in the group of women and by 12.5% in the group of men.

The reduction of FFM was most intense in the first three months after LSG (by 7.5% in the group of women and by 9.5% in the group of men). In both study groups, we observed a decrease in resting metabolic rate (RMR) already in the first month after the procedure, but it was statistically insignificant. A statistically significant decrease in RMR was observed only in the group of men 6 months after LSG.

In this study, we used the electrical bioimpedance method to assess body composition, which is not a precise method due to possible underestimation of body fat content and overestimation of FFM in morbidly obese patients [16,17]. However, we used this method because of the high cost and limited availability of computed tomography (CT) and dual-energy X-ray absorptiometry (DXA). It is possible that using CT or DXA to assess body composition would give more accurate results.

### 3.2. Blood Biochemical Parameters Analysis

The values of blood biochemical parameters of patients before and 1, 3, and 6 months after surgery and the percentage of patients with results above the reference values are presented in Table 2. In the group of men, in each of the assessed periods after surgery, fasting glucose and insulin concentrations were significantly lower than before the surgery. In the group of women, we observed a significant decrease in fasting glucose and insulin levels only 6 months after the procedure. Before the procedure, type 2 diabetes was present in 1 (6.6%) woman and 6 (40%) men. Six months after the surgery, we proved remission of diabetes in each of these patients. It should be noted, however, that 6 months after LSG, 40% of women and 60% of men still had abnormal fasting glucose. Moreover, in 20% of women and men, the HOMA-IR index was still ≥2.

During the 6-month follow-up, the serum concentration of TG and AST also significantly decreased in both study groups, and in the group of men, the serum concentration of ALT was also significantly reduced. The percentage of women with hypertriglyceridemia 6 months after the procedure decreased from 26.6% to 13.3% in comparison to the percentage before LSG, and from 60% to 20% in the group of men. The percentage of patients with hypercholesterolemia decreased in the group of women from 66.6% before to 33.3% 6 months after the surgery, and in the group of men from 66.6% to 40%. Mixed hyperlipidemia was present before the procedure in 20% of women and 53.3% of men. Within 6 months, this percentage decreased to 13.3% in both groups. On the other hand, the incidence of atherogenic dyslipidemia in the 6-month follow-up decreased in the group of men from 40% to 6.6%. In the group of women, atherogenic dyslipidemia occurred in 1 woman (6.6%) throughout the study period.

### 3.3. Dietary Assessment

Table 3 presents changes in the energy and nutritional value of diet in the periods assessed and the percentage of patients who did not meet the RDA or AI intake criteria. One, three, and six months after the surgery, the energy value of the diet of the subjects in both groups was, as expected, significantly lower compared to the supply before the surgery.

The median protein intake 1, 3, and 6 months after the surgery in the group of women and 1 and 3 months after the surgery in the group of men was lower than the recommended 60 g/d. One month after LSG, optimal protein intake was achieved by only 13.3% of women and 6.6% of men. Three months after the procedure it was achieved by only 20% of women and 33.3% of men, and six months after the procedure by 26.6% of women and 60% of men.

Insufficient intake of carbohydrates (at a level not exceeding 45% of energetic value of the diet) was found in 53.3% of women and 73.3% of men qualified for LSG. One month after the procedure, the intake of carbohydrates in both groups was too low in relation to the recommendations. Only 20% of patients achieved a carbohydrate intake of 50 g/d. Three months after the procedure, carbohydrate intake below the recommended 50 g/d was observed in 53.3% of women and 26.6% of men. Six months after surgery, the median intake of carbohydrates was 88.3 g/d in the group of women and 132.7 g/d in the group of men. Intake of carbohydrates below the recommendations (130 g/d) in this period was found in 66.6% of women and 46.6% of men. We also observed insufficient intake of dietary fiber in all women and 60% of men before the procedure, all women and men after 1 and 3 months, and in all women and 93.3% of men 6 months after the procedure.

The diet of patients before the operation was deficient, especially in calcium (80% of women and 53.3% of men), magnesium (73.3% of women and men), potassium (86.6% of women and 60% of men), folic acid (86.6% of women and 66.6% of men), and vitamin D (100% of women and 100% of men), and in the group of women, additionally in iron (93.3%). In both groups, the intake of minerals in each of the assessed periods after the operation (except for the intake of calcium in the group of women 3 and 6 months after the operation) was significantly lower than the intake supply before the operation.

Six months after LSG, the percentage of patients with insufficient intake of calcium, magnesium, and zinc in both study groups was 100%. The intake of iron and potassium was below the RDA/AI in all women and in 73.3% and 93.3% of men, respectively. Analyzing the intake of vitamins 6 months after the procedure, we observed an insufficient supply of vitamin B_1_ (100% of women and 86.6% of men), folates (100% of women and 100% of men), and vitamin D (100% of women and 93.6% of men). Moreover, the supply of vitamin B_6_ below the RDA was found in 73.3% of women and 46.6% of men, and in the case of vitamin B_12_, 66.6% of women and 40% of men.

## 4. Discussion

Laparoscopic sleeve gastrectomy is an effective method of surgical treatment of morbid obesity. In this study, we observed rapid weight loss in the first 6 months after surgery. EWL in the 6-month follow-up was 49.5% in the group of women and 47.6% in the group of men. A similar %EWL value was obtained by Adamczyk et al. [18]. In the study by Golzarand M. et al., the average EWL was higher and amounted to 66.4 ± 23.8% [19]. Similarly, in the study by Barzin M. et al., where EWL was 62.2 ± 15.7% [20]. On the other hand, the results published by Otto M. et al., indicate lower average EWL—39.1 ± 10.4% [21].

The results of our study, as well as those of other authors, indicate that the reduction of body weight after LSG was caused not only by the reduction of total body fat mass, but also by the reduction of fat free mass [7,8,19,20,22,23]. Six months after the procedure, we proved a reduction in FM by 37.5% in the group of women and by 37.06% in the group of men, as well as a reduction in FFM by 10% in the group of women and by 12.5% in the group of men. Belfiore et al. recorded an average reduction of 39% in FM in women and 40% in men, as well as a 10% decrease in FFM in women and 15.7% in men within 6 months after LSG [7]. Similar to our study, they found that the reduction of both, fat mass and fat free mass was most intense in the first three months after the surgery, after which the loss of fat free mass decelerated, while the mass of fat tissue continued to decrease [7]. Similar results have been obtained by other authors [10,18,20,24]. In the study by Otto et al., the reduction of fat mass, lean body mass, and body cell mass was most intense during the first 6 weeks after the procedure [21]. These observations are confirmed by Maïmoun L. et al., who report significant reduction in lean body mass in the first month after the surgery. Their study showed statistically insignificant differences between men and women [23].

It is known that the inevitable consequence of bariatric surgeries, including LSG, is a decrease in FFM. The loss of FFM, despite low energy supply, is undoubtedly also the result of insufficient protein intake in the diet. According to “Guidelines for Perioperative Care in Bariatric Surgery: Enhanced Recovery After Surgery (ERAS) Society Recommendations”, protein intake after surgical treatment of obesity should be at the level of 60–120 g/day [14]. On the other hand, according to the updated American clinical practice guidelines concerning, among other things, perioperative nutrition, a minimum daily protein intake of 60 g and a gradual increase to 1.5 g/kg of ideal body weight is recommended [15,25]. The results of studies by Moize et al. provide evidence that protein intake of >60 g/d or 1.1 g/kg of ideal body weight is connected with better maintenance of lean body mass (LBM) in patients after bariatric surgery [26].

In our study, only 13.3% of women and 6.6% of men achieved optimal protein intake one month after surgery. Three months after surgery, it was 20% of women and 33.3% of men, and 6 months after surgery, 26.6% of women and 60% of men. In the study by Dagan, protein intake > 60 g/d was achieved by only 15.1% of patients 3 months after the surgery and by 32.9% 6 months after the surgery [27]. Furthermore, in the study by Hirsch et al., protein intake > 60 g/d was achieved by a maximum of 79% of the subjects [28].

According to the available data, the average protein intake after LSG in particular periods after the surgery is at a very different level. One month after the procedure, the declared protein supply, depending on the study, ranged from 30.0 ± 15.9–70.4 ± 26.8 g/d. After 3 months, it was 34.9 ± 4.8–72.1 ± 23, 7 g/d, and after 6 months—36.7 ± 12.5–76.9 ± 15.2 g/d [8,9,19,28,29,30,31,32,33]. In our study, the median protein intake 1 month after the procedure was 31.4 g/d in the group of women and 32.6 g/d in the group of men, after 3 months—47.9 g/d and 51.5 g/d, respectively, and after 6 months—47.6 g/d and 67.9 g/d. The results of our studies, as well as those of the majority of other authors, indicate that protein intake in the first months after LSG is insufficient [8,9,22,27,29,30]. Undoubtedly, this has a negative impact on FFM. Dagan showed a statistically significant loss of FFM in 72% of patients consuming less than 60 g of protein per day. Protein intake > 60 g/d helps to preserve FFM and prevents its excessive loss [22,33,34]. According to Schiavo et al., protein intake at the level of 2 g/kg ideal body weight is more effective than protein intake at the level of 1.0 g/kg ideal body weight in reducing FM and is associated with lower FFM and RMR loss in patients after LSG [35].

The consequence of FFM loss is the activation of compensatory mechanisms including the increase of energy supply with food aimed at restoring FFM to the optimal level. This phenomenon is referred to as the so-called “collateral fattening” concept [36]. In the study by Sotiroux et al., decrease in FFM was positively correlated with decrease in basal metabolic rate (BMR) [37]. FFM loss is connected with weight regain due to decreased resting metabolic rate [38,39]. Decrease in resting metabolic rate and increase of energy supply are unfavorable for bariatric patients. It may lead to weight gain, and thus reduce the effectiveness of surgical treatment or cause the instability in its outcomes. In our study, in both study groups, decrease in resting metabolic rate (RMR) in each of the assessed periods after LSG was noted. It was statistically significant only in the group of men 6 months after the procedure.

Undoubtedly, determination of the optimal protein supply after bariatric surgery is problematic. Schollenberger suggests that protein supplementation contributes to the increase in the loss of body fat mass and decreases in the loss of lean body mass over in 6-month observation [33]. Therefore, it seems reasonable to include high-protein preparations in the diet, especially in the first 3 months after LSG, in order to cover the demand for this nutrient and thus minimize the loss of lean body mass.

Intensive body weight reduction in patients after LSG is caused by insufficient energy intake in relation to body energy demand [7,8,9,10,22,23,30,31]. Kanverva et al. suggest that long-term postoperative weight loss is not only the result of energy deficit, but may also be related to the ratio of macronutrients. They recommend reducing the intake of fat with the diet by <35% of diet energy value in favor of proteins (20%) and carbohydrates (≥45%) [40]. These guidelines coincide with the “Clinical practice guidelines for the perioperative nutrition, metabolic, and nonsurgical support of patients undergoing bariatric procedures” [25].

Carbohydrates are energy material for the brain and muscles. According to recommendations for the Polish population, they should cover 45–65% of energy value of the diet. In our study, the percentage share of carbohydrates in covering the energy value of the diet of patients qualified for LSG was too low—it was 42.6% in the group of women and 41.6% in the group of men. This conforms with the results of other authors [13,22,26,27,41].

According to recommendations in the early postoperative period, patients should intake at least 50 g of carbohydrates per day [15]. This amount should gradually increase to 130 g/d in the later period [15]. Observational studies have shown that the average intake of carbohydrates in the first 6 months after LSG is at the level of 38.6–109 g/d, which is 23–46% of the energy value of the diet [9,10,19,29,30,41]. In our study, we also observed an insufficient intake of carbohydrates. The median carbohydrate intake 1 month after the surgery in both groups was too low in relation to the recommendations and amounted to 37.7 g/d in the group of women and 32.6 g/d in the group of men. Only 20% of the subjects reached the minimum intake of carbohydrates. Three months after the surgery, the recommended intake of at least 50 g/d was found only in 46.6% of women and 73.3% of men. Six months after the procedure, as many as 66.6% of women and 46.6% of men did not achieve the recommended carbohydrate intake of ≥130 g/d. The median share of carbohydrates in the energy value of the diet 1 month after LSG was 33.4% in the group of women and 37% in the group of men. Six months after the surgery, it increased to 47.9% and 49.7%, respectively.

Attention should also be paid to the intake of fiber in the diet of patients after LSG. In our study and in the study of Coluzzi et al., it was insufficient both before and after the surgery [30]. Due to the common problem of constipation, patients after LSG should gradually increase the consumption of vegetables, fruits, and whole grains, as well as increasing fluid intake. In case of long-term constipation, supplementation with a water-soluble fiber preparation should be considered [42].

Recommendations for fat intake after bariatric surgery are similar to those for the general population. Fat should cover 20–35% of daily caloric intake, with the majority being unsaturated fatty acids [15]. In our study, the percentage of fat covering the energy value of the daily diet before the surgery was 36.2% in the group of women and 36.5% in the group of men. The percentage of fat in the energy content of the diet was 24.2–24.4% after 1 month, 24.5–28.9% after 3 months and 24.8–25.1% 6 months after LSG. The results of other studies indicate higher consumption of fats, exceeding 35% of the total energy supply [9,10,19,22,30,41]. It should be noted that in our study, the share of unsaturated fatty acids in the diet was insufficient, because approximately 50% of the consumed fats were saturated fatty acids. These observations are confirmed by Batar et al. [8,10].

In our study, we found that the diet of obese patients who qualified for LSG was deficient in calcium (80% of women and 53.3% of men), magnesium (73.3% of women and men), potassium (86.6% of women and 60% of men), folic acid (86.6% of women and 66.6% of men), and vitamin D (100% of women and 100% of men), and, in the group of women, iron (93.3%). In the study by Dagan et al., supplies of iron, calcium, folic acid, vitamin B_12_, and vitamin B_1_ below the Dietary Reference Intake (DRI) was reported in 46%, 48%, 58%, 14%, and 34% of patients, respectively [6]. Insufficient intake of calcium, iron, folates, magnesium, and vitamin D in patients qualified for SG was also shown by Sanchez [43]. In many publications, the most frequently diagnosed deficiencies in patients qualified for SG were deficiencies of vitamin D (22–90% of patients), iron (15–51% of patients), vitamin B_12_ (11.5–16% of patients), zinc (11.5–30% of patients), and magnesium (approximately 38% of patients) [5,6,7,8,9,10].

Six months after LSG, deficiencies of vitamin D, vitamin B_6_, and zinc are the main diagnoses [7,9,41]. In addition, deficiencies of folic acid, vitamin B_1_ and iron may occur [7,9,41]. It is connected with the fact that the diet of SG patients is lacking in vitamins and minerals. In our study, in each of the assessed periods after the operation, the intake of vitamins and minerals was significantly reduced. Six months after the surgery, in both study groups, the median intake of calcium, iron, magnesium, potassium, zinc, vitamins B_1_, D, and folate, and in the group of women additionally B_6_, B_12_ was below the RDA or AI. Similar results were obtained by Batar, who reports that 6 months after LSG. The intake of vitamins (A, E, C, B_1_, B_12_, folate) and minerals (calcium, magnesium, iron, zinc, phosphorus, and iodine) was below the Dietary Reference Intake (DRI) recommendations [8]. Furthermore, Moize observed insufficient intake of calcium, magnesium, potassium, and iron, compared to the DRI, 6 months after SG [41]. In the study by Wawrzyniak and Krotki, insufficient intake of calcium, potassium, magnesium, sodium, and zinc was found in 97%, 97%, 83%, 60%, and 53% of SG patients, respectively, and in the group of women iron in 50%. It should be noted that in the abovementioned study, the intake of minerals was given as the average value from all three stages (i.e., 3, 6, and 9 months after SG) for each patient. Mean intakes of calcium, magnesium, iron, and zinc were compared to the Estimated Average Requirement (EAR) [44].

Insufficient intake of vitamins and minerals with the diet results in their deficiencies in the body which, if not compensated for, will deepen. As a consequence, many complications may develop, including polyneuropathy, metabolic bone disease, anemia, and Wernicki–Kosrakov disease [45,46].

Supplementation is a key element in compensating for and preventing the development of nutritional deficiencies and their complications. In the study by Verger et al., the percentage of patients with nutritional deficiencies 6 months after the surgery decreased in relation to the percentage of patients with deficiencies diagnosed before surgery. It is connected to the fact that before the SG, only 10% of patients systematically took vitamin and mineral supplements, while within 3 months after the procedure, this percentage increased to 77% [10]. However, as reported by Belfiore, taking standard multivitamin preparations was effective in correcting deficiencies in about 30% of patients [7]. It was noted that deficiencies of vitamin B_1_ and zinc did not respond to supplementation with multivitamin preparations, which may indicate that they contain insufficient amounts of those nutrients [7].

A small percentage of patients undergoing laparoscopic sleeve gastrectomy may experience early post-operative complications, such as gastric leak. These patients require stomach decompression and restriction of oral feeding. In case oral feeding is restricted, nutrition should be maintained by a feeding jejunostomy, naso-jejunal feeding, or parenteral nutrition [47,48].

Body weight reduction and regression of obesity-related co-morbidities, quality of life improvement, and reduction of the risk of death are arguments in favor of surgical treatment of morbid obesity [49]. In our study, in the 6-month follow-up, we showed improvement in the clinical condition of patients undergoing SG. In both groups, fasting glucose and insulin concentrations as well as TG and AST concentrations were significantly reduced and in the group of men, ALT and CRP concentrations decreased.

The improvement of glucose and insulin metabolism was visible already within the 3rd month after the surgery. Our observations are confirmed by the results of the study by Guillet et al. and Dagan et al., who reported a reduction in fasting glucose and insulin levels, triglycerides, and total cholesterol 3 months after SG [27,50]. Batar et al., 6 months after SG, reported statistically significant decreases in fasting insulin and glucose, TC, LDL, TG, ALT, AST, and increases in HDL in the group of women and men [8].

Sleeve gastrectomy (SG) improves glycemic control and initiates diabetes remission in 95–100% of patients [51]. In meta-analysis by Yip et al., remission of type 2 diabetes was demonstrated in 56.3% of patients 3 months after SG. This rate increased to 68% 12 months after the SG [52]. In our study, we also observed remission of type 2 diabetes.

In the study by Milone et al., 3 months after SG, in addition to changes in BMI, glycemia, and HbA1C, a significant reduction in total cholesterol, LDL-cholesterol, and triglycerides, accompanied by an increase in HDL-cholesterol were also observed. After 6 months, further favorable changes in the values of the aforementioned parameters were achieved [53]. In our study, 6 months after the procedure, we also showed a reduction in the percentage of patients with lipid disorders. According to the authors, SG may be the preferred method of surgical treatment of obesity in patients with dyslipidemia [53].

The decrease of triglycerides, ALT, and AST concentrations in blood serum undoubtedly indicate an improvement in the functioning of the liver. In one of the studies, in 1-year follow-up, it was shown that a decrease in the concentration of ALT, AST, and γ-GT in blood serum was correlated with the remission of non-alcoholic fatty liver disease [54]. The percentage of patients with fatty liver decreased from 84.4% before surgery to 10.8% 12 months after SG. This is confirmed by the results of the study by Bower, who showed that bariatric surgery is associated with a significant improvement in biochemical and histological markers of non-alcoholic fatty liver disease [55].

## 5. Conclusions

Laparoscopic sleeve gastrectomy is highly effective in reducing excessive body mass. Undoubtedly, it also improves the glucose, insulin, and lipid metabolism of patients operated on. However, it should be emphasized that an extremely important element of the therapy of patients with obesity treated surgically is the optimization of nutrition. We have shown that 6 months after surgery the intake of protein, calcium, magnesium, zinc, iron, folate, vitamin B_1_, and vitamin D was below the RDA. In light of this, it seems crucial to ensure an adequate supply of protein and supplementation of vitamins and minerals in order to prevent nutritional deficiencies and their complications.

## Figures and Tables

**Table 1:** Changes in body composition 1, 3, and 6 months after LSG in the group of women and men.

			Median		
Parameter		Before Surgery	1st Month	3rd Month	6th Month
Weight (kg)	F	119.0	109.4 *	103.0 **	91.00 ***
	M	150.0	139.0	123.5 **	106.00 ***
EWL (%)	F	-	17.4	30.4	49.5 ^†^
	M	-	16.0	31.4	47.6 ^†^
BMI (kg/m^2^)	F	43.7	39.1 *	36.0 **	32.7 ***
	M	40.7	42.0	38.5 **	34.2 ***
Total body fat mass (kg)	F	58.9	50.3	44.4 **	36.2 ***
	M	69.2	60.3	48.1 **	37.9 ***
Fat free mass (kg)	F	58.8	55.7	53.9 **	53.6 ***
	M	84.0	75.8	75.5 **	72.5 ***
% of total body fat mass loss	F	-	13.5	22.9	37.3 ^†^
	M	-	9.9	21.0	37.0 ^†^
% of fat free mass loss	F	-	5.2	7.5	10.0 ^†^
	M	-	6.1	9.5	12.5 ^†^
RMR (kcal/d)	F	1655.0	1619.0	1619.0	1616.0
	M	2345.0	2193.0	2151.0	2088.0 ***

* *p* < 0.05 difference between 1st month and before surgery; ** *p* < 0.05 difference between 3rd month and before surgery; *** *p* < 0.05 difference between 6th month and before surgery; ^†^ *p* < 0.05 difference between %EWL 6th month and 1st month; BMI - body mass index; F—female; M—men; % EWL—percentage of excess weight loss; RMR—resting metabolic rate.

**Table 2:** The values of blood biochemical parameters of women and men before and 1, 3, and 6 months after LSG and the percentage of patients with results above the reference values.

	Reference Values	Median				% of Patients above the References Values			
		Before Surgery	1st Month	3rd Month	6th Month	Before	1st Month	3rd Month	6th Month
FPG (mg/dl)	F: 70–99	105.0	100.0	100.0	97.0 ***	73.3	60.0	53.3	40.0
	M: 70–99	116.0	102.0 *	103.0 **	100.0 ***	93.3	46.6	53.3	60.0
FPI (mIU/l)	F: 3–17	12.4	7.9	6.8 **	5.7 ***	33.3	13.3	6.6	6.6
	M: 3–17	22.6	11.5 *	9.5 **	6.8 ***	73.3	26.6	20.0	6.6
HOMA-IR	F: <2	2.6	1.8	1.5 **	1.4 ***	73.3	46.6	33.3	20.0
	M: <2	6.8	3.0 *	2.3 **	1.6 ***	100	86.6	80.0	20.0
TC (mg/dl)	F: <190	206.0	174.0	194.0	183.0	73.3	33.3	53.3	40.0
	M: <190	213.0	187.0	184.0	187.0	66.6	33.3	40.0	46.6
LDL (mg/dl)	F: < 115	126.0	110.0 *	112.5	120.1	73.3	33.3	46.6	60.0
	M: <115	131.7	118.5	117.2	115.6	73.3	60.0	60.0	46.6
HDL (mg/dl)	F: >50	47.0	41.0	45.0	57.0	53.3	73.3	53.3	40.0
	M: >40	40.0	35.0 *	40.0	42.0	46.6	73.3	60.0	46.6
TG (mg/dl)	F: <150	124.0	99.0	97.0	88.0 ***	26.6	6.6	13.3	13.3
	M: <150	151.0	120.0	124.0	109.0 ***	60	26.6	33.3	20.0
AST (U/I)	F: 5–34	22.0	25.0	21.0	17.0 ***	13.3	26.6	0	13.3
	M: 5–34	27.5	27.6	23.0	22.4 ***	33.3	20.0	13.3	0
ALT (U/I)	F: 0–55	30.0	31.0	22.0	18.0 ***	6.6	0	0	20.0
	M: 0–55	51.70	40.5	36.0	22.4	20.0	20.0	6.6	0

* *p* < 0.05 difference between 1st month and before surgery; ** *p* < 0.05 difference between 3rd month and before surgery; *** *p* < 0.05 difference between 6th month and before surgery; Me—median; %—percentage of patients above the references values (HDL—below the references values); F—female; M—men; FPG—Fasting plasma glucose, FPI—Fasting plasma insulin; HOMA-IR - homeostasis model assessment of insulin resistance; TC—total cholesterol; LDL—low density lipoprotein; HDL—high density lipoprotein; TG—triglycerides; AST—Aspartate aminotransferase; ALT—Alanine aminotransferase.

**Table 3:** Energetic value of the diet and selected nutrient intake before, 1, 3, and 6 months after LSG in the group of women and men and percentage of patients below the RDA/AI.

Nutrient	RDA/AI	Median				% of Patients Below the RDA/AI			
		Before Surgery	1st Month	3rd Month	6th Month	Before	1st Month	3rd Month	6th Month
Energy (kcal/d)	F	1766.2	453.1 *	519.0 **	766.2 ***				
	M	2726.6	397.4 *	632.4 *	990.5 ***				
Protein (g/d)	F: ≥60	73.2	31.4 *	47.9 **	47.6 ***	0	86.6	80.0	73.3
	M: ≥60	129.6	32.6 *	51.5 *	67.9 ***	0	93.3	66.6	40.0
Carbohydrates (g/d)	F: 50 or 130	231.9	37.7 *	48.4 **	88.3 ***	13.3	80.0	53.3	66.6
	M: 50 or 130	264.5	32.6 *	65.4 **	132.7 ***	0	80.0	26.6	46.6
Fibre (g/d)	F: ≥25	17.2	2.4 *	5.8 **	7.7 ***	100	100	100	100
	M: ≥25	19.6	2.6 *	6.6 **	7.7 ***	60.0	100	100	93.3
Ca (mg/d)	F: 18	553.2	354.7 *	462.6	397.0	80.0	100	100	100
	M: 10	924.0	243.0 *	485.4 **	516.1 ***	53.3	100	86.6	100
Fe (mg/d)	F: 320	10.2	2.0 *	3.1 **	5.0 ***	93.3	100	100	100
	M: 420	15.2	2.4 *	3.8 **	6.5 ***	13.3	100	93.3	73.3
Mg (mg/d)	F: 4700	264.5	84.5 *	122.8 **	172.4 ***	73.3	100	100	100
	M: 4700	378.4	84.5 *	176.4 **	197.1 ***	73.3	100	100	100
K (mg/d)	F: 8	3212.6	1205.8 *	1528.0 **	1784.2 ***	86.6	100	100	100
	M: 11	1966.6	956.7 *	1841.0 **	2285.6 ***	60.0	100	100	93.3
Zn (mg/d)	F: 1.1	9.2	3.0 *	3.7 **	4.2 ***	40.0	100	100	100
	M: 1.3	15.5	2.8 *	4.2 **	6.5 ***	20.0	100	100	100
Wit. B_1_ (mg/d)	F: 1.1	1.2	0.3 *	0.4 **	0.5 ***	33.3	100	100	100
	M: 1.3	2.0	0.2 *	0.3 **	0.7 ***	13.3	100	100	86.6
Wit. B_6_ (mg/d)	F: 1.3	1.6	0.7 *	0.9 **	1.0 ***	26.6	100	86.6	73.3
	M: 1.3	2.7	0.7*	1.0 **	1.3 ***	13.3	93.3	86.6	46.6
Folates (µg/d)	F: 400	279.8	67.2 *	110.3 **	127.8 ***	86.6	100	100	100
	M: 400	341.5	83.1 *	123.5 **	176.5 ***	66.6	100	100	100
Wit. B_12_ (µg/d)	F: 2.4	3.0	1.7 *	1.8	2.0	40.0	66.6	53.3	66.6
	M: 2.4	4.4	1.9 *	2.2 **	3.6	20.0	73.3	60.0	40.0
Wit. D (µg/d)	F: 15	2.4	0.2 *	1.0 **	1.0	100	100	100	100
	M: 15	2.8	0.7 *	1.0 **	1.1	100	100	100	93.3

* *p* < 0.05 difference between 1st month and before surgery; ** *p* < 0.05 difference between 3rd month and before surgery; *** *p* < 0.05 difference between 6th month and before surgery; Me—median; F—female; M—men; Ca—calcium; Fe—iron; Mg—magnesium; K—potassium; Zn—zinc.

## Data Availability

Data available on request due to restrictions, e.g., privacy or ethical.

## References

[1] https://www.who.int/news-room/fact-sheets/detail/obesity-and-overweight.

[2] Centrum Badań Opinii Publicznej Komunikat z Badań. Czy Polacy Mają Problem z Nadwagą?. https://www.cbos.pl/SPISKOM.POL/2019/K_103_19.PDF.

[3] Obesity Rate by Body Mass Index (BMI). https://ec.europa.eu/eurostat/databrowser/view/sdg_02_10/default/table?lang=en.

[4] Chang S.H., Stoll C.R.T., Song J., Varela J.E., Eagon C.J., Colditz G.A. (2014). The effectiveness and risks of bariatric surgery an updated systematic review and meta-analysis, 2003–2012. JAMA Surg..

[5] Al-Mutawa A., Anderson A.K., Alsabah S., Al-Mutawa M. (2018). Nutritional status of bariatric surgery candidates. Nutrients.

[6] Dagan S.S., Zelber-Sagi S., Webb M., Keidar A., Raziel A., Sakran N., Goitein D., Shibolet O. (2016). Nutritional status prior to laparoscopic sleeve gastrectomy surgery. Obes. Surg..

[7] Belfiore A., Cataldi M., Minichini L., Aiello M.L., Trio R., Rossetti G., Guida B. (2015). Short-term changes in body composition and response to micronutrient supplementation after laparoscopic sleeve gastrectomy. Obes. Surg..

[8] Batar N., Demir H.P., Bayram H.M. (2021). Assessment of nutritional status, body composition and blood bio-chemical parameters of patients following sleeve gastrectomy: 6 months follow up. Clin. Nutr. ESPEN.

[9] Vinolas H., Barnetche T., Ferrandi G., Monsaingeon-Henry M., Pupier E., Collet D., Gronnier C., Gatta-Cherifi B. (2019). Oral hydration, food intake, and nutritional status before and after bariatric surgery. Obes. Surg..

[10] Verger E.O., Aron-Wisnewsky J., Dao M.C., Kayser B.D., Oppert J.M., Bouillot J.L., Torcivia A., Clément K. (2016). Micronutrient and protein deficiencies after gastric bypass and sleeve gastrectomy: A 1-year follow-up. Obes. Surg..

[11] Lim H.S., Kim Y.J., Lee J., Yoon S.J., Lee L. (2020). Establishment of adequate nutrient intake criteria to achieve target weight loss in patients undergoing bariatric surgery. Nutrients.

[12] Nahler G. (2017). Dictionary of Pharmaceutical Medicine.

[13] Jarosz M., Rychlik E., Stoś E., Charzewska J. (2020). Normy Żywienia dla Populacji Polski i ich Zastosowanie.

[14] Thorell A., MacCormick A.D., Awad S., Reynolds N., Roulin D., Demartines N., Vignaud M., Alvarez A., Singh P.M., Lobo D.N. (2016). Guidelines for perioperative care in bariatric surgery: Enhanced Recovery after Surgery (ERAS) Society Recommendations. World J. Surg..

[15] Kushner R.F., Herron D.H., Herrington H. Bariatric Surgery: Postoperative Nutritional Management. UpToDate. https://www.uptodate.com/contents/bariatric-surgery-postoperative-nutritional-management?search=Bariatric%20surge-ry:%20postoperative%20nutritional%20management&source=search_result&selectedTitle=1~150&usage_type=default&display_rank=1.

[16] Deurenberg P. (1996). Limitations of the bioelectrical impedance method for the assessment of body fat in severe obesity. Am. J. Clin. Nutr..

[17] Leal A.A., Faintuch J., Morais A.A., Noe J.A., Bertollo D.M., Morais R.C., Cabrini D. (2011). Bioimpedance analysis: Should it be used in morbid obesity?. Am. J. Hum. Biol..

[18] Adamczyk P., Bužga M., Holéczy P., Švagera Z., Šmajstrla V., Zonča P., Pluskiewicz W. (2015). Bone mineral density and body composition after laparoscopic sleeve gastrectomy in men: A short-term longitudinal study. Int. J. Surg..

[19] Golzarand M., Toolabi K., Djafarian K. (2019). Changes in body composition, dietary intake, and substrate oxidation in patients underwent laparoscopic roux-en-Y gastric bypass and laparoscopic sleeve gastrectomy: A comparative prospective study. Obes. Surg..

[20] Barzin M., Almasi M.H., Mahdavi M., Khalaj A., Valizadeh M., Hosseinpanah F. (2021). Body composition changes following sleeve gastrectomy vs. one-anastomosis gastric bypass: Tehran Obesity Treatment Study (TOTS). Obes. Surg..

[21] Otto M., Elrefai M., Krammer J., Weiß C., Kienle P., Hasenberg T. (2016). Sleeve gastrectomy and roux-en-Y gastric bypass lead to comparable changes in body composition after adjustment for initial body mass index. Obes. Surg..

[22] Dagan S.S., Tovim T.B., Keidar A., Raziel A., Shibolet O., Zelber-Sagi S. (2017). Inadequate protein intake after laparoscopic sleeve gastrectomy surgery is associated with a greater fat free mass loss. Surg. Obes. Relat. Dis..

[23] Maïmoun L., Lefebvre P., Jaussent A., Fouillade C., Mariano-Goulart D., Nocca D. (2017). Body composition changes in the first month after sleeve gastrectomy based on gender and anatomic site. Surg. Obes. Relat. Dis..

[24] Friedrich A.E., Damms-Machado A., Meile T., Scheuing N., Stingel K., Basrai M., Küper M.A., Kramer K.M., Königsrainer A., Bischoff S.C. (2013). Laparoscopic sleeve gastrectomy compared to a multidisciplinary weight loss program for obesity—Effects on body composition and protein status. Obes. Surg..

[25] Mechanick J.I., Apovian C., Brethauer S., Garvey W.T., Joffe A.M., Kim J., Kushner R.F., Lindquist R., Pessah-Pollack R., Seger J. (2020). Guidelines Clinical practice guidelines for the perioperative nutrition, metabolic, and nonsurgical support of patients undergoing bariatric procedures—2019 update: Cosponsored by American Association of Clinical Endocrinologists/American College of Endocrinology, The Obesity Society, Ame-rican Society for Metabolic & Bariatric Surgery, Obesity Medicine Association, and American Society of Anesthesio-logists. Surg. Obes. Relat. Dis..

[26] Moizé V., Andreu A., Rodríguez L., Flores L., Ibarzabal A., Lacy A., Jiménez A., Vidal J. (2013). Protein intake and lean tissue mass retention following bariatric surgery. Clin. Nutr..

[27] Dagan S.S., Keidar A., Raziel A., Sakran N., Goitein D., Shibolet O., Zelber-Sagi S. (2017). Do bariatric patients follow dietary andlifestyle recommendations during the first postoperative year?. Obes. Surg..

[28] Hirsch K.R., Blue M.N.M., Trexler E.T., Ahuja S., Smith-Ryan A.E. (2021). Provision of ready-to-drink protein following bariatric surgery: An evaluation of tolerability, body composition, and metabolic rate. Clin. Nutr..

[29] Bertoni L., Valentini R., Zattarin A., Belligoli A., Bettini S., Vettor R., Foletto M., Spinella P., Busetto L. (2021). Assessment of protein intake in the first three months after sleeve gastrectomy in patients with severe obesity. Nutrients.

[30] Coluzzi I., Raparelli L., Guarnacci L., Paone E., Del Genio G., le Roux C.W., Silecchia G. (2016). Food intake and changes in eating behavior after laparoscopic sleeve gastrectomy. Obes. Surg..

[31] Palacio A.C., Quintiliano D., Vargas P., Cosentino M., Ríos M.J. (2021). Calorie and macronutrient in-take during the first six months after bariatric surgery. Rev. Méd. Chile..

[32] Ziadlou M., Hosseini-Esfahani F., Khosravi H.M., Hosseinpanah F., Barzin M., Khalaj A., Valizadeh M. (2020). Dietary macro- and micro-nutrients intake adequacy at 6th and 12th month post-bariatric surgery. BMC Surg..

[33] Schollenberger A.E., Karschin J., Meile T., Küper M.A., Königsrainer A., Bischoff S.C. (2016). Impact of protein supplementation after bariatric surgery: A randomized controlled double-blind pilot study. Nutrition.

[34] Leidy H.J., Clifton P.M., Astrup A., Wycherley T.P., Westerterp-Plantenga M.S., Luscombe-Marsh N.D., Wo-Ods S.C., Mattes R.D. (2015). The role of protein in weight loss and maintenance. Am. J. Clin. Nutr..

[35] Schiavo L., Scalera G., Pilone V., De Sena G., Quagliariello V., Iannelli A., Barbarisi A. (2017). A comparative study examining the impact of a protein-enriched vs normal protein postoperative diet on body composition and resting metabolic rate in obese patients after sleeve gastrectomy. Obes. Surg..

[36] Dulloo A.G., Jacquet J., Miles-Chan J.L., Schutz Y. (2017). Passive and active roles of fat-free mass in the control of energy intake and body composition regulation. Eur. J. Clin. Nutr..

[37] Sotirxou M., Migdanis A., Migdanis I., Kanaki M., Koukoulis G., Alexandrou A., Diamantis T. (2016). Changes in body composition and basic metabolic rate (BMR) following bariatric surgry. Clin. Nutr. ESPEN.

[38] Vink R.G., Roumans N.J., Arkenbosch L.A., Mariman E.C., van Baak M.A. (2016). The effect of rate of weight loss on long-term weight regain in adults with overweight and obesity. Obesity.

[39] Hopkins M., Finlayson G., Duarte C., Whybrow S., Ritz P., Horgan G.W., Blundell J.E., Stubbs R.J. (2016). Model-ling the associations between fat-free mass, resting metabolic rate and energy intake in the context of total energy balance. Int. J. Obes..

[40] Kanerva N., Larsson I., Peltonen M., Lindroos A.K., Carlsson L.M. (2017). Changes in total energy intake and ma-cronutrient composition after bariatric surgery predict long-term weight outcome: Findings from the Swedish Obese Subjects (SOS) study. Am. J. Clin. Nutr..

[41] Moizé V., Andreu A., Flores L., Torres F., Ibarzabal A., Delgado S., Lacy A., Rodriguez L., Vidal J. (2013). Long-term dietary intake and nutritional deficiencies following sleeve gastrectomy or Roux-en-Y gastric bypass in a mediterranean population. J. Acad. Nutr. Diet..

[42] Dagan S.S., Goldenshluger A., Globus I., Schweiger C., Kessler Y., Sandbank G.K., Ben-Porat T., Sinai T. (2017). Nutritional recommendations for adult bariatric surgery patients: Clinical practice. Adv. Nutr..

[43] Sánchez A., Rojas P., Basfi-Fer K., Carrasco F., Inostroza J., Codoceo J., Valencia A., Papapietro K., Csen-des A., Ruz M. (2016). Micronutrient deficiencies in morbidly obese women prior to bariatric surgery. Obes. Surg..

[44] Wawrzyniak A., Krotki M. (2021). The need and safety of mineral supplementation in adults with obesity post bariatric surgery—Sleeve gastrectomy (SG). Obes. Surg..

[45] Argyrakopoulou G., Konstantinidou S.K., Dalamaga M., Kokkinos A. (2022). Nutritional deficiencies before and after bariatric surgery: Prevention and treatment. Curr. Nutr. Rep..

[46] Lupoli R., Lembo E., Saldalamacchia G., Avola C.K., Angrisani L., Capaldo B. (2017). Bariatric surgery and long-term nutritional issues. World J. Diabetes.

[47] Lazzarin G., Di Furia M., Romano L., Di Sibio A., Di Giacomo C.C., Lombardi L., Giuliani A., Schietroma M., Pessia B., Carlei F.F. (2020). Endoscopic Double-Pigtail Catheter (EDPC) Internal Drainage as First-Line Treatment of Gastric Leak: A Case Series during Laparoscopic Sleeve Gastrectomy Learning Curve for Morbid Obesity. Minim. Invasive Surg..

[48] Praveenraj P., Gomes R.M., Kumar S., Senthilnathan P., Parthasarathi R., Rajapandian S., Palanivelu C. (2016). Management of gastric leaks after laparoscopic sleeve gastrectomy for morbid obesity: A tertiary care experience and design of a management algorithm. J. Minim. Access Surg..

[49] Fuchs T., Loureiro M., Both G.H., Skraba H.H., Costa-Casagrande T.A. (2017). The role of the sleeve gastrectomy and the management of type 2 diabetes. Arq. Bras.Cir. Dig..

[50] Guillet C., Masgrau A., Mishellany-Dutour A., Blot A., Caille A., Lyon N., Pereira B., Slim K., Robert M., Disse E. (2020). Bariatric surgery affects obesity-related protein requirements. Clin. Nutr. ESPEN.

[51] Stefater M.A., Inge T.H. (2017). Bariatric surgery for adolescents with type 2 diabetes: An emerging therapeutic strategy. Curr. Diabetes Rep..

[52] Yip S., Plank L.D., Murphy R. (2013). Gastric bypass and sleeve gastrectomy for type 2 diabetes: A systematic review and meta-analysis of outcomes. Obes. Surg..

[53] Milone M., Lupoli R., Maietta P., Di Minno A., Bianco P., Ambrosino P., Coretti G., Milone F., Di Minno M.N., Musella M. (2015). Lipid profile changes in patients undergoing bariatric surgery: A comparative study between sleeve gastrectomy and mini-gastric bypass. Int. J. Surg..

[54] Guan B., Chen Y., Chong T.H., Peng J., Mak T.K., Wang C., Yang J. (2020). Effect of bariatric surgery on serum enzyme status in obese patients. Obes. Surg..

[55] Bower G., Toma T., Harling L., Jiao L.R., Efthimiou E., Darzi A., Athanasiou T., Ashrafian H. (2015). Bariatric surgery and non-alcoholic fatty liver disease: A systematic review of liver biochemistry and histology. Obes. Surg..

